# Blood pressure and mortality after percutaneous coronary intervention: a population-based cohort study

**DOI:** 10.1038/s41598-022-06627-4

**Published:** 2022-02-17

**Authors:** Chung-woo Lee, Joo Kyung Lee, Yeon Joo Choi, Hyunjin Kim, Kyungdo Han, Jin-hyung Jung, Do Hoon Kim, Joo-Hyun Park

**Affiliations:** 1 Department of Family Medicine, Korea University Ansan Hospital, College of Medicine, Korea University, 123, Jeokgeum-ro, Danwon-gu, Ansan-si, Gyeonggi-do 15355 South Korea; 2 Department of Internal Medicine, Korea University Guro Hospital, Korea University College of Medicine, Seoul, Republic of Korea; 3 Department of Statistics and Actuarial Science, Soongsil University, Seoul, Republic of Korea; 4 Department of Biostatistics, College of Medicine, The Catholic University of Korea, Seoul, Republic of Korea

**Keywords:** Acute coronary syndromes, Hypertension, Cardiology, Risk factors

## Abstract

Revascularization procedures, including percutaneous coronary intervention (PCI), for coronary artery disease (CAD) are increasingly performed in Korea. However, studies on blood pressure control targets in these patients remain insufficient. To assess the relationship between baseline blood pressure and all-cause mortality in CAD patients who underwent PCI. A population-based retrospective cohort study based on the national claims database of the Korean National Health Insurance System, which represents the entire Korean population. A total 38,330 patients with a history of PCI for CAD between 2005 and 2008 were recruited and followed up for all-cause mortality until December 31, 2017. Baseline systolic blood pressure (SBP) and diastolic blood pressure (DBP) were measured, and they were classified into eight SBP and DBP groups each. The hazard ratios (HRs) for all-cause mortality were measured for each group. The pattern of SBP and DBP in this population followed a J-curve relationship for all-cause mortality, with the nadir point at 119 and 74 mmHg, respectively. In subjects aged > 60 years, high SBP (≥ 160 mmHg) and high DBP (≥ 90 mmHg) were significantly related to death. Moreover, in subjects aged > 60 years, low DBP (< 70 mmHg) was significantly related to mortality. There is a J-curve relationship between baseline blood pressure and all-cause mortality in patients who underwent PCI, and intensive lowering of blood pressure may be beneficial for these patients. However, the elderly population needs more attention as excessive BP lowering, particularly DBP, could instead increase the risk of death.

## Introduction

Cardiac diseases are one of the major causes of death in Korea. In 2017, 60.2 deaths per 100,000 Koreans were associated with heart-related problems, resulting in the second highest mortality rate^1^. The rate of revascularization for coronary artery diseases (CADs) has been steadily increasing. In addition, the demand for percutaneous coronary intervention (PCI) and coronary artery bypass graft surgery has increased in 2006–2017, from 40,035 to 74,993 cases. Among the interventions performed in 2017, a total of 71,037 of 74,993 cases were PCI procedures^2^. Thus, the importance of management for CAD in patients with a PCI history is currently receiving growing attention. Especially as a major risk factor for CAD, hypertension should be managed for preventing the progression of diseases in this population^3^.

The optimal BP target in patients with CAD is not yet clear^4–6^. Recently published guidelines in the United States, Europe, and Korea recommend the target systolic blood pressure (SBP) as 130 mmHg and diastolic blood pressure (DBP) as 80 mmHg in CAD patients^3,7–9^. Despite the guideline recommendations, the ideal BP target in patients with a CAD history remains controversial, with the recent emergence of the new breakthrough hypothesis about the existence of a J-curve relationship between the degree of blood pressure (BP) control and its outcomes^3^. In previous studies, J-curve relationships between BP and outcomes, such as all-cause mortality and myocardial infarction (MI) incidence in CAD patients, have been reported^4,5,10–19^. However, most of these results were from post-hoc analyses of previous studies, and it is difficult to reflect real-world situations. Therefore, further research is needed to determine the accurate and ideal degree of BP reduction for CAD patients. Nonetheless, there are insufficient data and published studies on BP and its relation to other outcomes in CAD patients who have survived after coronary artery revascularization procedures such as PCI.

This study aimed to identify the best BP targets in this population by investigating the relationship between the degree of BP control and all-cause mortality in patients who underwent PCI for CAD. Thus, we analyzed the relationship between baseline BP level and death of patients who underwent PCI by using the Korean National Health Insurance System (NHIS) database.

## Methods

### Data and subjects

In this nationwide cohort study, we used data from the Korean NHIS database. The Korean NHIS provides general health screening examinations to all Korean citizens every 2 years for the prevention and early detection of diseases^20^. The NHIS database consists of comprehensive medical data, including diagnoses using the International Classification of Disease 10th revision (ICD-10) codes, procedures, prescription medications, demographics, and personal information^21^. The study population included Korean patients who underwent general health screening between 2005 and 2008 and had a history of undergoing PCI for CAD within 2 years prior to the screening examination. A total 38,330 subjects were eligible for inclusion in the study. From the general health screening they were followed up for all-cause mortality until December 31, 2017.

Data are available from the Korea National Health Insurance Sharing Service Institutional Data Access/Ethics Committee (https://nhiss.nhis.or.kr/bd/ay/bdaya001iv.do) for researchers who meet the criteria for access to confidential data. Researchers can apply for the National Health Insurance data sharing service upon approval of the Institutional Review Board of their institution. After review of the Korea National Health Insurance Sharing Service Institutional Data Access/Ethics Committee, authors are required to pay a data access fee and confirm that other researchers will be able to access the data in the same manner as the authors. The study was conducted according to the ethical principles outlined in the Declaration of Helsinki. This study was approved by the institutional review board of Korea University Ansan Hospital (2017AS0437). Due to retrospective nature of the study the informed consent was waived by ethics committee of Korea University Ansan Hospital. All data were anonymous and de-personalized.

### Definitions

The blood pressure was measured by automatic or digital monitors after resting for at least 5 min in sitting position during the general health screening for the subjects between 2005 and 2008. And the blood pressure was set as the baseline BP. The baseline BP was further stratified into eight subgroups in increments of 10 mmHg for SBP (< 100, 100–109, 110–119, 120–129, 130–139, 140–149, 150–159, and ≥ 160 mmHg) and seven subgroups in increments of 10 mmHg for DBP (< 60, 60–69, 70–79, 80–89, 90–99, 100–109, and ≥ 100 mmHg). The subjects’ history of PCI was identified using the procedure codes of the Korean Health Insurance Review and Assessment Service. Procedure codes corresponding to PCI are defined as M6551–M6554, M6561–M6566, M6571, and M6572.

The outcome measure of this study was the first occurrence of death of any cause. To determine the baseline characteristics of the subjects, covariate data that could affect the mortality of CAD patients were collected. The covariate data are as followed: age (< 60 years, ≥ 60 years), sex, smoking (non-smoker, ex-smoker, current smoker), alcohol intake (none; mild, less than 30 g/day; heavy, more than 30 g/day), exercise, yearly income, body mass index (BMI). Lifestyle data were based on answers provided by subjects in standardized questionnaires conducted upon their national health screening.

Diabetes was determined based on fasting blood glucose levels of ≥ 126 mg/dL measured during the health screening or based on ICD-10 codes (E11-E14) along with registered anti-diabetic drug prescriptions, and dyslipidemia with total cholesterol levels ≥ 240 mg/dL, or ICD-10 code E78 and lipid lowering drug prescriptions.

### Statistical analysis

The baseline characteristics of subjects were classified into continuous and categorical variables. The incident rates are described as number of events per 1000 person-years. The hazard ratios (HRs) and 95% confidence intervals (CIs) were estimated using Cox proportional models to analyze the relationship between BP values and mortality rates in subjects with a PCI history. Covariates (age, sex, lifestyle factors, BMI, and comorbidities) were entered into the models for adjustment. Two-tailed P values < 0.05 were considered statistically significant. And we extrapolated spline curves of adjusted HRs with 95% CIs, according to SBP and DBP. Statistical analyses were conducted using SAS version 9.4 (SAS Institute Inc., Cary, NC, USA).

## Results

The mean age of the study population was 62.0 years, with 41.7% aged ≥ 60 years and 58.3% aged ≤ 60 years. The study population consisted of mostly men, accounting for 72.4%. Surprisingly, more than half of the participants were non-smokers (64.9%) and non-alcohol consumers (71.2%). Considering the medical history, 31.4% of the subjects had diabetes mellitus, 87.0% had hypertension, and 84.5% had dyslipidemia. Overweight was defined as BMI ≥ 23 kg/m^2^ and obesity was defined as BMI ≥ 25 kg/m^2^, according to the obesity guidelines in Korea^22^. Our population consisted of 28.6% overweight and 44.7% obese subjects. The mean SBP was 127.8 mmHg and the mean DBP was 77.8 mmHg (Table 1).

**Table 1:** Baseline characteristics of the study population.

	All (N = 38,330)
Mean age (years)	62.0
**Age group (%)**	
< 60 years	58.3
≥ 60 years	41.7
Male sex (%)	72.4
**Smoking status (%)**	
Non-smoker	64.9
Ex-smoker	19.1
Current	13.6
**Alcohol intake**^**a**^** (%)**	
No	71.2
Mild	9.6
Heavy	14.2
Diabetes mellitus^b^ (%)	31.4
Hypertension^c^ (%)	87.0
Dyslipidemia^d^ (%)	84.5
Abdominal obesity (%)	53.2
**BMI**^**e**^** (%)**	
< 23 kg/m^2^	26.7
≥ 23 and < 25 kg/m^2^	28.6
≥ 25 and < 30 kg/m^2^	40.5
≥ 30 kg/m^2^	4.2
Mean BMI^e^ (kg/m^2^)	25.0
Mean SBP^f^ (mmHg)	127.8
Mean DBP^g^ (mmHg)	77.8

^a^None; mild, < 30 g/day; heavy, > 30 g/day.

^b^Fasting blood glucose levels of ≥ 126 mg/dL measured during the health screening, or based on ICD-10 codes (E11–E14) along with registered anti-diabetic drug prescriptions.

^c^One or more diagnostic codes of ICD-10 I10–13 or I15 and prescription of antihypertensive drugs.

^d^Total cholesterol levels ≥ 240 mg/dL, or ICD-10 code E78 and prescriptions of lipid-lowering drugs.

^e^Body mass index; B*ody weight (kg)* divided by the square of the *body* height (m).

^f^Systolic blood pressure.

^g^Diastolic blood pressure.

**Figure 1:**
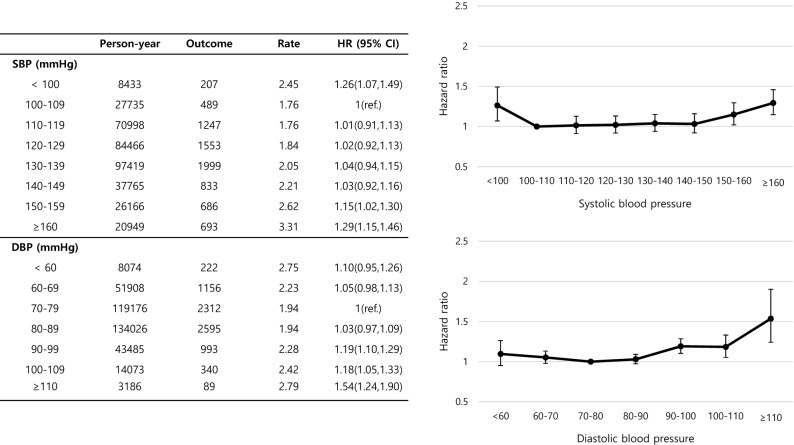
Hazard ratio (HR) for all-cause mortality according to systolic blood pressure (SBP) and diastolic blood pressure (DBP) groups. Data were adjusted for age, sex, body mass index (BMI), lifestyle, and presence of diabetes mellitus (DM) and dyslipidemia. The 95% confidence intervals (CIs) are shown as vertical lines with the hazard ratios (HRs).

The effect of BP on these subjects who had undergone PCI, and the relationship between the BP subgroups and the HRs are demonstrated in Fig. 1. The reference interval for SBP was 100–109 mmHg and that for DBP was 70–79 mmHg. The groups with SBP < 100 mmHg (HR 1.26, 95% CI 1.07–1.49) and ≥ 150 mmHg (SBP 150–159; HR 1.15, 95% CI 1.02–1.30, SBP ≥ 160; HR 1.29, 95% CI 1.15–1.46) and DBP 90–110 mmHg (DBP 90–99; HR 1.19, 95% CI 1.10–1.29, DBP 100–109; HR 1.18, 95% CI 1.05–1.33, DBP ≥ 110; HR 1.54, 95% CI 1.24–1.90) were significantly associated with the highest HRs, displaying a U-shaped pattern for SBP and more of a J-shaped relationship for DBP.

Table 2 shows the number of mortality events, follow-up duration, and death rate per 1000 person-years among the SBP and DBP subgroups. The reference interval for SBP was 100–109 mmHg and that for DBP was 70–79 mmHg. For subjects younger than 60 years, SBP < 100 mmHg (HR 1.417, 95% CI 1.043–1.927) and ≥ 150 mmHg (HR 1.346, 95% CI 1.062–1.706 for SBP < 160 mmHg and HR 1.908, 95% CI 1.504–2.421 for SBP ≤ 160 mmHg) showed a significant association with death rates, with a J-curve pattern. The lowest and highest SBP subgroups presented significance. In addition, the DBP in this age group did not show any significance. Among subjects aged > 60 years, the high SBP and DBP groups, with SBP ≥ 160 mmHg (HR 1.158, 95% CI 1.008–1.33) and DBP ≥ 90 mmHg, were significantly related to death. In addition, among subjects aged > 60 years, the low DBP group (< 70 mmHg) was significantly related to mortality. When sex was considered for significance, SBP < 100 mmHg (HR 1.3, 95% CI 1.075–1.572) and ≥ 150 mmHg (HR 1.214, 95% CI 1.056–1.398 for SBP < 160 mmHg and HR 1.417, 95% CI 1.23–1.633 for SBP ≤ 160 mmHg) in men showed a J-curve pattern of HRs.

**Table 2:** Effect of blood pressure on all-cause mortality by subgroup.

	Events	Duration	Rate (%)	HR (95% CI)
**Age < 60 years**				
SBP (mmHg)	n			
< 100	60	5595.5	1.07	1.42 (1.04–1.93)
100–109	146	19,502.5	0.75	1 (ref.)
110–119	400	48,767.8	0.82	1.12 (0.92–1.36)
120–129	455	54,788.6	0.83	1.15 (0.95–1.39)
130–139	482	58,576.1	0.82	1.11 (0.92–1.34)
140–149	180	19,941.1	0.90	1.13 (0.90–1.42)
150–159	146	13,146.5	1.11	1.35 (1.06–1.71)
≥ 160	145	8666.6	1.67	1.91 (1.50–2.42)
DBP (mmHg)	n			
< 60	47	4193.4	1.12	1.10 (0.81–1.49)
60–69	273	30,749.5	0.89	0.95 (0.82–1.10)
70–79	656	74,173.4	0.88	1 (ref.)
80–89	686	84,244.5	0.81	0.93 (0.84–1.04)
90–99	238	25,654.4	0.93	1.05 (0.90–1.22)
100–109	89	8030.0	1.11	1.16 (0.92–1.46)
≥ 110	25	13,162.2	1.29	1.43 (0.95–2.15)
**Age ≥ 60 years**				
SBP (mmHg)	n			
< 100	147	2837.1	5.18	1.21 (1.00–1.48)
100–109	343	8232.8	4.17	1 (ref.)
110–119	847	22,229.8	3.81	0.97 (0.86–1.10)
120–129	1098	29,677.2	3.70	0.97 (0.86–1.10)
130–139	1517	38,843	3.91	1.01 (0.89–1.13)
140–149	653	17,824.1	3.66	0.99 (0.87–1.14)
150–159	540	13,019.7	4.15	1.08 (0.94–1.24)
≥ 160	548	12,282.4	4.46	1.16 (1.01–1.33)
DBP (mmHg)	n			
< 60	175	3880.3	4.51	1.10 (0.94–1.30)
60–69	883	21,158.8	4.17	1.09 (1.01–1.19)
70–79	1656	45,003.0	3.68	1 (ref.)
80–89	1909	49,781.9	3.83	1.07 (1.00–1.14)
90–99	755	17,831.5	4.23	1.25 (1.14–1.36)
100–109	251	6043.4	4.15	1.20 (1.04–1.37)
≥ 110	64	1247.4	5.13	1.57 (1.22–2.02)
**Male sex**				
SBP (mmHg)	n			
< 100	161	6294.5	2.56	1.30 (1.08–1.57)
100–109	374	21,397.0	1.75	1 (ref.)
110–119	950	54,635.8	1.74	1.05 (0.93–1.19)
120–129	1141	63,463.0	1.80	1.05 (0.94–1.19)
130–139	1445	70,021.9	2.06	1.08 (0.96–1.22)
140–149	573	25,340.3	2.26	1.09 (0.95–1.24)
150–159	468	16,793.5	2.79	1.22 (1.06–1.40)
≥ 160	445	11,881.9	3.75	1.42 (1.23–1.63)
DBP (mmHg)	n			
< 60	160	5442.3	2.94	1.08 (0.91–1.28)
60–69	872	37,600.0	2.32	1.07 (0.99–1.17)
70–79	1689	87,383.9	1.93	1 (ref.)
80–89	1871	98,037.5	1.91	1.04 (0.97–1.16)
90–99	689	30,099.6	2.29	1.21 (1.11–1.33)
100–109	222	9219.9	2.41	1.23 (1.06–1.42)
≥ 110	54	2044.8	2.64	1.51 (1.15–1.99)
**Female sex**				
SBP (mmHg)	n			
< 100	46	2138.2	2.15	1.12 (0.79–1.59)
100–109	115	6338.3	1.81	1 (ref.)
110–119	297	16,361.7	1.82	0.91 (0.73–1.13)
120–129	412	21,002.8	1.96	0.92 (0.75–1.14)
130–139	554	27,397.2	2.02	0.93 (0.76–1.14)
140–149	260	12,424.9	2.09	0.90 (0.72–1.12)
150–159	218	9372.7	2.33	1.00 (0.79–1.26)
≥ 160	248	9067.2	2.74	1.05 (0.84–1.31)
DBP (mmHg)	n			
< 60	62	2631.3	2.36	1.13 (0.87–1.47)
60–69	284	14,308.3	1.99	0.99 (0.86–1.15)
70–79	623	31,792.5	1.96	1 (ref.)
80–89	724	35,988.8	2.01	1.01 (0.90–1.12)
90–99	304	13,386.3	2.27	1.14 (0.99–1.31)
100–109	118	4853.4	2.43	1.10 (0.90–1.35)
≥ 110	35	1142.1	3.06	1.57 (1.12–2.21)
**Without DM**				
SBP (mmHg)	n			
< 100	135	6038.4	2.24	1.32 (1.08–1.63)
100–109	295	20,086.7	1.47	1 (ref.)
110–119	826	51,400.4	1.61	1.09 (0.95–1.25)
120–129	925	59,272.3	1.56	1.01 (0.89–1.16)
130–139	1191	67,537.7	1.76	1.04 (0.91–1.18)
140–149	472	25,259.4	1.87	1.04 (0.90–1.21)
150–159	393	17,010.4	2.31	1.16 (0.99–1.35)
≥ 160	371	13,282.4	2.79	1.24 (1.06–1.45)
DBP (mmHg)	n			
< 60	131	5316.3	2.46	1.16 (0.96–1.40)
60–69	683	35,543.5	1.92	1.05 (0.95–1.15)
70–79	1393	83,403.6	1.67	1 (ref.)
80–89	1557	94,188.8	1.65	1.01 (0.94–1.10)
90–99	588	29,815.7	1.97	1.18 (1.07–1.30)
100–109	206	9514.3	2.17	1.21 (1.04–1.41)
≥ 110	50	2105.6	2.37	1.47 (1.11–1.96)
**With DM**				
SBP (mmHg)	n			
< 100	72	2394.3	3.01	1.16 (0.87–1.53)
100–109	194	7648.6	2.54	1 (ref.)
110–119	421	19,597.1	2.15	0.89 (0.75–1.06)
120–129	628	25,193.4	2.49	1.02 (0.87–1.20)
130–139	808	29,881.5	2.70	1.04 (0.89–1.22)
140–149	361	12,505.8	2.89	1.02 (0.85–1.22)
150–159	293	9155.8	3.20	1.14 (0.94–1.37)
≥ 160	322	7666.6	4.20	1.34 (1.12–1.61)
DBP (mmHg)	n			
< 60	91	2757.3	3.30	1.04 (0.83–1.29)
60–69	473	16,364.8	2.89	1.06 (0.95–1.19)
70–79	919	35,772.7	2.57	1 (ref.)
80–89	1038	39,837.6	2.61	1.06 (0.97–1.16)
90–99	405	13,670.2	2.96	1.20 (1.07–1.36)
100–109	134	4559.1	2.94	1.14 (0.95–1.37)
≥ 110	39	1081.3	3.61	1.64 (1.19–2.26)

*CI* confidence interval, *DBP* diastolic blood pressure, *DM* diabetes mellitus, *HR* hazard ratio, *SBP* systolic blood pressure.

**Figure 2:**
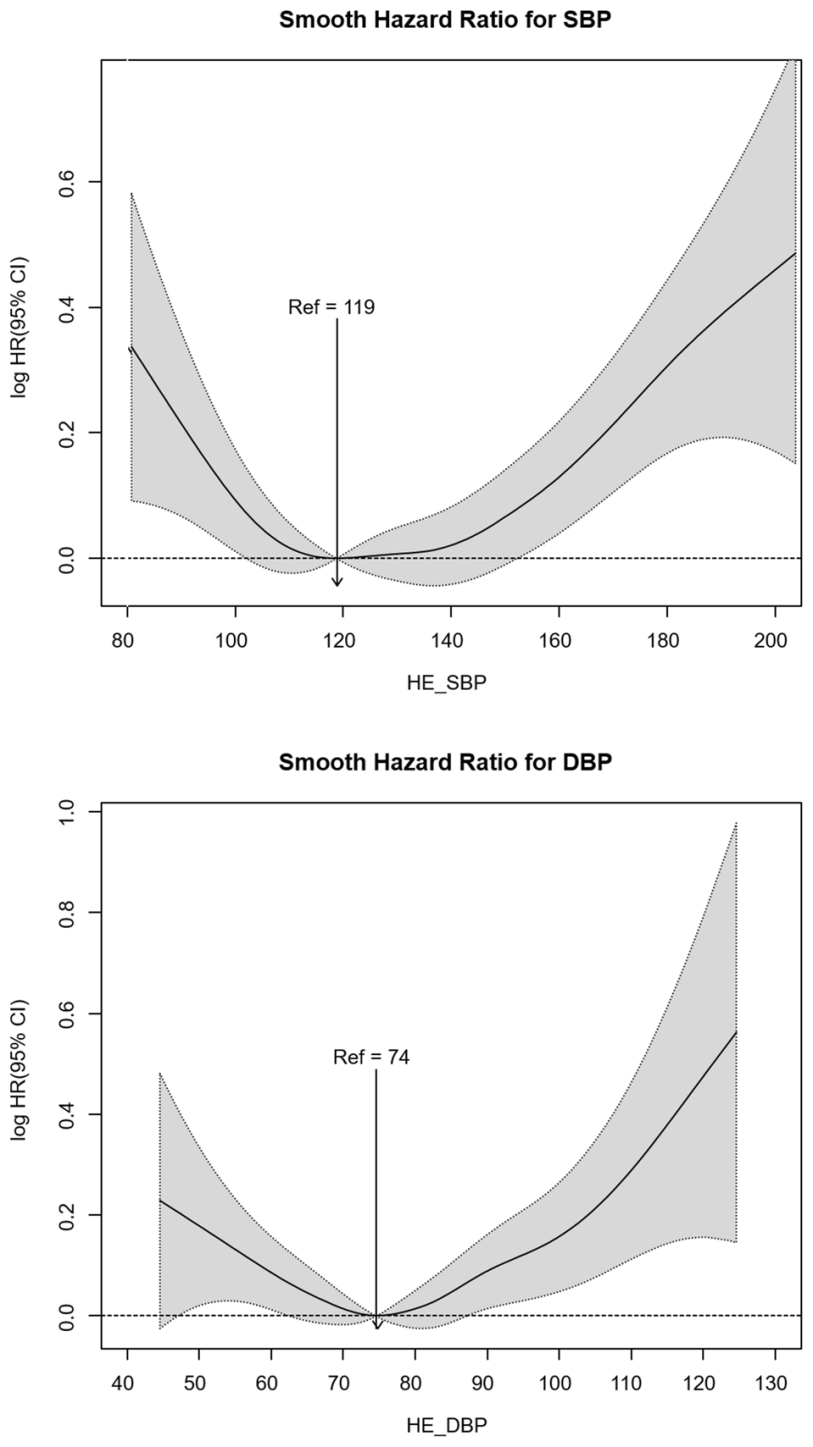
Spline curves of mooth hazard ratio (HR) for all-cause mortality according to systolic blood pressure (SBP) and diastolic blood pressure (DBP).

By summing up the data discussed above, the nadir BP point could be set as 119/74 mmHg. The HR is more likely to steeply increase in both directions for SBP 119 mmHg. For DBP > 74 mmHg, the HR more sharply increased than values < 74 mmHg (Fig. 2).

## Discussion

In this study of Korean subjects who underwent PCI for CAD, it was found that the relationship between BP and all-cause mortality follows a J-shaped relationship in terms of both SBP and DBP. And the identified nadir BP point that was related to the lowest all-cause mortality in this study was 119/74 mmHg, which was lower than that of the value recommended in preexisting guidelines. This study suggests that intensive BP lowering is effective in this patient population, but it is necessary to appropriately control BP especially when considering age, because of the high risk of mortality from excessive BP lowering. As there is a high risk of mortality from low DBPs in elderly patients, it is important to apply a less strict target for diastolic BP reduction in this population.

### Interpretation and comparison with other studies

In this study of Korean subjects who underwent PCI for CAD, it was found that the relationship between BP and all-cause mortality follows a J-shaped relationship. Similar results have been indicated in previous studies in CAD patients; however, the identified nadir BP point that was related to the lowest all-cause mortality in this study was 119/74 mmHg, which is lower than the latest known value. In the Treating to New Targets trial, the BP nadir (the nadir BP value found to be associated with the lowest incidence of death due to CAD and non-fatal MI) was 146.3/81.4 mmHg^13^. The analysis of patients in the PROVE IT-TIMI (PRavastatin Or atorVastatin Evaluation and Infection Therapy-Thrombolysis In Myocardial Infarction) trial showed that the BP nadir, which had the lowest incidence of primary outcomes such as all-cause mortality and MI, was 136/85 mmHg^14^. In the analysis of patients who underwent coronary revascularization among those who participated in INVEST (INternational VErapamil SR-trandolapril STudy), the BP nadir, which had the lowest prevalence of primary outcomes such as all-cause mortality and non-fatal MI, was 145/80 mmHg in the PCI-treated group^17^. In the ONTARGET (ONgoing Telmisartan Alone and in combination with Ramipril Global Endpoint Trial), the SBP nadir, which had the lowest percentage of primary outcomes such as cardiovascular death, MI, and stroke, was about 130 mmHg^12^. Overall, the nadir BP proved to have the lowest prevalence of primary outcomes in previous studies tended to be higher than that in our study. On the contrary, in the analysis of patients who participated in INVEST, the BP nadir that had the least occurrence of all-cause death, non-fatal MI, and non-fatal stroke was 119/84 mmHg, similar to the results of this study^23^.

In particular, as shown in recent studies, intensive BP lowering that targets an SBP of < 120 mmHg may have a positive effect on the long-term survival of CAD patients who had undergone PCI^24,25^. In addition, this study showed that antihypertensive administration for BP control in patients with a PCI history who had BP below the hypertension criterion could have survival benefits. This is consistent with the results of a previous meta-analysis showing that lowering BP through antihypertensive treatment may be beneficial in patients with a cardiovascular disease history but without hypertension^26^.

It is particularly noteworthy that when the subjects were divided by age group, there was a difference in the relationship between BP and all-cause mortality in young adults aged < 60 and > 60 years. Younger adults with SBP between 100 and 110 mmHg tended to have the lowest mortality, whereas those aged > 60 years had the lowest mortality when SBP was between 120 and 130 mmHg. This is similar to the results of a previous study in patients with CAD whose SBP values with the lowest all-cause mortality, cardiovascular mortality, MI, and other outcomes increased in the elderly population^4,16^. It is also consistent with the existing guidelines recommending relatively less strict BP targets for elderly hypertensive patients^8,9^.

### Implications for research and practice

The debate between “the lower the better” hypothesis and the “J-curve phenomenon” hypothesis has long persisted in setting BP control goals^11,27^. Many studies have shown that mortality and cardiovascular adverse events increase at high and low BP values. Nevertheless, previous meta-analyses and the recent SPRINT study have shown that intensive BP lowering to SBP < 120 mmHg positively affects mortality and other outcomes^24,25,28^. This supports “the lower the better” hypothesis in BP, which also influenced the guidelines published by the American College of Cardiology and the American Heart Association in 2017^7^. The results of this study showed a high prevalence of all-cause mortality in both the low SBP and DBP groups, suggesting that there is a J-curve relationship between BP and death in CAD patients who had undergone PCI. At the same time, the results of this study also suggest that intensive BP lowering targeting an SBP of < 120 mmHg may have a survival benefit for CAD patients who had undergone PCI. It is important to consider that the actual rate of BP control by antihypertensive medication is only about 70% in Korean patients in 2016^29^. Therefore, physicians in the clinical field should not hesitate to actively control the BP of patients who had undergone PCI.

Although there is a J-curve relationship between blood pressure and all-cause mortality, the CAD patients who underwent PCI can benefit from intensive BP lowering. In this study, we covered only all-cause mortality as outcome. In addition to all-cause mortality, studies are needed to investigate cardiovascular outcomes such as recurrent MI and stroke.

### Strengths and limitations of this study

This study has some limitations. First, this study analyzed the results of one-time measurement of BP during the subjects’ health examination. Therefore, this study does not reflect the fluctuation of BP values and, consequently, the measurement of BP may be inaccurate. Second, as the subjects were patients who had undergone a medical examination after undergoing PCI for CAD, there is a risk of selection bias in that patients who were relatively healthy and who were merely interested in health care may have been included. Third, the occurrence of cause-specific mortality or any other related cardiovascular event could have be ignored because only all-cause mortality was calculated as the outcome. Fourth, this study targeted those who underwent PCI within 2 years of receiving a medical checkup. Therefore, there is a limitation in that the interval between the time when PCI was performed and the time when BP was measured among the subjects of this study is not constant, which may have affected the results. Nevertheless, this study examined the incidence of mortality in CAD patients who survived after PCI, as well as obtained specific results and provided important insights into the management of this particular patient population. Moreover, the risk of reverse causality was reduced by excluding patients who died early after PCI and by adjusting for various factors including lifestyle and underlying diseases. This study also has strength in that the average follow-up period was at least 7 years, providing powerful evidence for the long-term management of CAD survivors.

In conclusion, the results of this study in CAD patients who underwent PCI suggests that the mortality was the lowest in BP corresponding to intensive BP lowering in this patient population. Although there is a J-curve relationship between BP and all-cause mortality, the CAD patients who underwent PCI can benefit from intensive BP lowering. This study only covered all-cause mortality as outcome. In addition to all-cause mortality, studies are needed to investigate cardiovascular outcomes such as recurrent MI and stroke.

## Abbreviations

BMI: Body mass index
BP: Blood pressure
CAD: Coronary artery disease
CI: Confidence interval
DBP: Diastolic blood pressure
HR: Hazard ratio
ICD-10: International Classification of Disease 10th revision
INVEST: INternational VErapamil SR-trandolapril Study
MI: Myocardial infarction
NHIS: National Health Insurance System
PCI: Percutaneous coronary intervention
PROVE IT-TIMI: PRavastatin Or atorVastatin Evaluation and Infection Therapy-Thrombolysis In Myocardial Infarction
SBP: Systolic blood pressure
SPRINT: Systolic Blood Pressure Intervention Trial
